# Extrapulmonary Tuberculosis Presenting With Double Vision in a Resource-Limited Tropical Setting

**DOI:** 10.4269/ajtmh.18-0473

**Published:** 2019-01

**Authors:** Anna Blum, Junior Mudji, Johannes Blum

**Affiliations:** 1 Hôpital Evangélique de Vanga, Bandundu, Democratic Republic of Congo;; 2 Swiss Tropical and Public Health Institute, Basel, Switzerland;; 3 Medical Faculty, University of Basel, Basel, Switzerland

**Figure 1.**
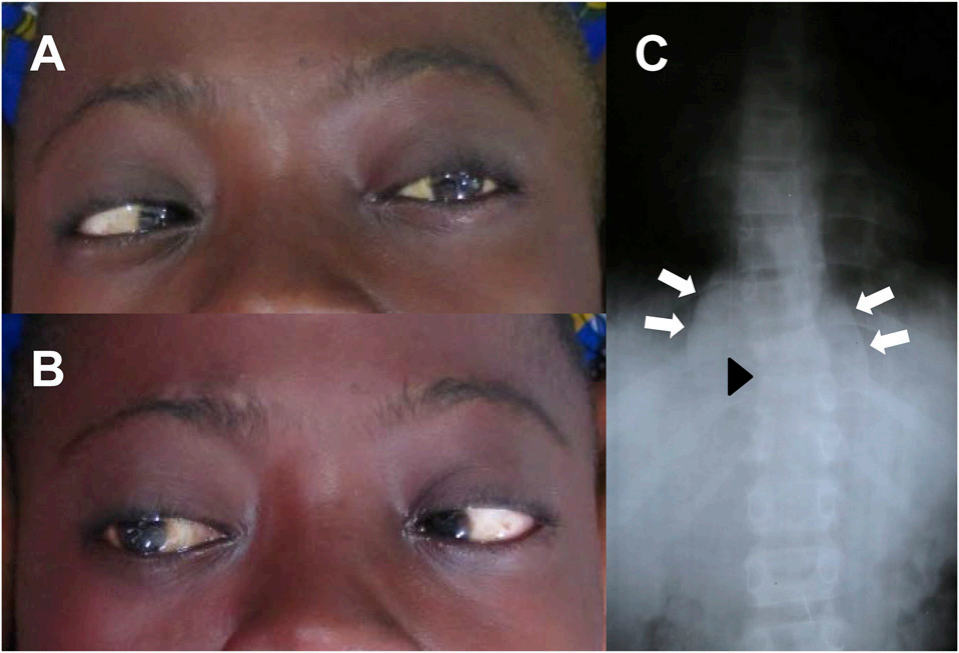
(**A** and **B**) bilateral abducens palsy as a sign for tuberculous basal meningitis and (**C**) anterior spondylodiscitis at T 10-11 with a paravertebral abscess (Pott‘s disease). This figure appears in color at www.ajtmh.org.

A 16-year-old boy was admitted to a rural hospital in the Democratic Republic of Congo with a 1-month history of abdominal pain, headache, and nightly fevers, and a 1-week history of seizures. Physical examination included general weakness, low-grade fever (37.5°C), neck rigidity, and bilateral abducens palsy leading to double vision (Figure 1A and B), typical clinical findings consistent with tuberculous basal meningitis.^1^ The latter was supported by lumbar puncture showing white blood cells of 720 cells/mm^3^ (90% mononuclear) and elevated protein (Pandy test positive) but negative Ziehl-Neelsen stain. In 20% of meningeal tuberculosis cases, the cerebrospinal fluid cell count is between 500 and 1,500/mm^3^, and in 65% between 100 and 500/mm^3^.^1^

In addition, physical examination revealed spinal tenderness at T 10-11 with normal reflexes, sensation, and strength of lower extremities bilaterally. Abdominal and cardiopulmonary examination was unremarkable. Spinal X-ray showed destructive anterior spondylodiscitis at T 10-11 with a paravertebral abscess (Figure 1C), again typical findings for Pott’s disease^2^ and explaining the abdominal pain. Simultaneous pulmonary tuberculosis or human immunodeficiency virus (HIV) infection were ruled out by chest X-ray and a HIV rapid test.

Due to pulmonary tuberculosis already experienced as an infant and current extrapulmonary manifestation, an extended treatment scheme with rifampicin, isoniazid, ethambutol, pyrazinamide, and streptomycin (2SRHZE/RHZE/9RH), as well as initially beginning with corticosteroids (prednisone 1 mg/kg) was chosen.

Within 2 weeks, the abducens palsy was resolved completely, the abdominal pain improved, and the spinal fluid cell count dropped to 23 cells/mm^3^. One month later, the patient was back to normal daily life, playing football with friends.

Tuberculosis is still endemic in many low- and middle-income countries such as the Democratic Republic of Congo with a tuberculosis incidence of 323 cases per year per 100,000 inhabitants and a high number of undetected cases every year.^3^

This case illustrates that a good clinical examination, knowledge on the epidemiological background, and basic laboratory technology may allow diagnosis and successful treatment even without modern technologies such as polymerase chain reaction and magnetic resonance imaging/computed tomography scan in resource-limited settings.
